# Supplementary material for: Global Mental Health and Services for Migrants in Primary Care Settings in High-Income Countries: A Scoping Review
Source: Int J Environ Res Public Health. 2020 Nov 20;17(22):8627. doi: 10.3390/ijerph17228627 (PMC7699722; doi:10.3390/ijerph17228627)
Supplement: Supplementary file 1 [file ijerph-17-08627-s001.zip › Additional file 2.pdf]

Search Strategy for MEDLINE

1. Refugees/
2. refugee*.mp.
3. (Displaced adj2 (person* or people* or population*)).mp.
4. (asylum adj2 seek*).mp.
5. Undocumented Immigrants/
6. ((undocumented or illegal or irregular) adj2 (immigrant* or migrant* or worker)).mp.
7. 1 or 2 or 3 or 4 or 5 or 6
8. Primary Health Care/
9. primary health care.mp.
10. primary healthcare.mp.
11. primary care.mp.
12. primary medical care.mp.
13. Community Health Services/
14. (community adj2 (care or health or healthcare or service*)).mp.
15. Physicians, Family/
16. Family physician*.mp.
17. Family doctor*.mp.
18. General Practitioners/
19. General practitioner*.mp.
20. GPs.mp.
21. GP.mp.
22. Nurse Practitioners/
23. Nurse practitioner*.mp.
24. Patient Care Team/
25. Family health team*.mp.
26. Shared care model*.mp.
27. 8 or 9 or 10 or 11 or 12 or 13 or 14 or 15 or 16 or 17 or 18 or 19 or 20 or 21 or 22 or 23 or 24 or 25 or 26

28. Mental Health/

29. mental health.mp.

30. exp Mental Disorders/

31. mental disorder*.mp.

32. psychiatric disease*.mp.

33. psychiatric disorder*.mp.

34. psychiatric illness*.mp.

35. Stress Disorders, Post-Traumatic/

36. ptsd.mp.

37. post-traumatic stress disorder.mp.

38. posttraumatic stress disorder.mp.

39. mental illness*.mp.

40. Anxiety Disorders/

41. anxiety.mp.

42. anxiety disorder*.mp.

43. Depressive Disorder, Major/

44. depression.mp.

45. 28 or 29 or 30 or 31 or 32 or 33 or 34 or 35 or 36 or 37 or 38 or 39 or 40 or 41 or 42 or 43 or 44

46. 7 and 27 and 45

47. limit 46 to yr="2000 -Current"
